# Plant Litter Type Dictates Microbial Communities Responsible for Greenhouse Gas Production in Amended Lake Sediments

**DOI:** 10.3389/fmicb.2018.02662

**Published:** 2018-11-06

**Authors:** Kurt M. Yakimovich, Erik J. S. Emilson, Michael A. Carson, Andrew J. Tanentzap, Nathan Basiliko, Nadia C. S. Mykytczuk

**Affiliations:** ^1^Vale Living with Lakes Centre, Laurentian University, Sudbury, ON, Canada; ^2^Department of Plant Sciences, University of Cambridge, Cambridge, United Kingdom; ^3^Department of Biology, Laurentian University, Sudbury, ON, Canada; ^4^Natural Resources Canada, Great Lakes Forestry Centre, Sault Ste. Marie, ON, Canada; ^5^School of the Environment, Laurentian University, Sudbury, ON, Canada

**Keywords:** bacteria, fungi, decomposition, lake sediments, anaerobic, methane, plant litter decomposition, methanogen

## Abstract

The microbial communities of lake sediments play key roles in carbon cycling, linking lakes to their surrounding landscapes and to the global climate system as incubators of terrestrial organic matter and emitters of greenhouse gasses, respectively. Here, we amended lake sediments with three different plant leaf litters: a coniferous forest mix, deciduous forest mix, cattails (*Typha latifolia*) and then examined the bacterial, fungal and methanogen community profiles and abundances. Polyphenols were found to correlate with changes in the bacterial, methanogen, and fungal communities; most notably dominance of fungi over bacteria as polyphenol levels increased with higher abundance of the white rot fungi *Phlebia* spp. Additionally, we saw a shift in the dominant orders of fermentative bacteria with increasing polyphenol levels, and differences in the dominant methanogen groups, with high CH_4_ production being more strongly associated with generalist groups of methanogens found at lower polyphenol levels. Our present study provides insights into and basis for future study on how shifting upland and wetland plant communities may influence anaerobic microbial communities and processes in lake sediments, and may alter the fate of terrestrial carbon entering inland waters.

## Introduction

Freshwater ecosystems have an important role in the global carbon (C) cycle because they can offset the terrestrial C sink by an estimated 79% via CO_2_ and CH_4_ emissions (Bastviken et al., 2011). A large portion of the C cycled through freshwater ecosystems comes from plants, with an estimated 2.9 Pg of terrestrial C year^-1^ entering freshwaters from terrestrial systems (three times what oceans receive) (Tranvik et al., 2009). Earth’s ∼304 million lakes are big players in C-cycling, harboring sediment microbial communities responsible for decomposing and mineralizing terrestrially derived C, under the form of organic matter (OM) (Downing et al., 2006). Most lakes are small, with organic-rich sediments that support obligately anaerobic CH_4_ production. Evasion of CH_4_ to the atmosphere can be accelerated by processes such as plant mediated efflux, ebullition, and wave action disturbance of the sediments (Hofmann et al., 2010), which highlights the importance of small lakes for overall lake CH_4_ budgeting (Bastviken et al., 2008). Additionally with CH_4_ being ∼25 times more potent a greenhouse gas than CO_2_ over a 100 yr timeframe, it becomes important to understand how microbial decomposers direct terrestrial C into CH_4_ (Forster et al., 2007).

Terrestrially derived C differs in biogeochemical composition from within-lake sources of OM (Field et al., 1988; Wetzel, 1992; Ye et al., 2016), however, little is known about how these differences influence sediment microbial decomposer communities (Hoostal and Bouzat, 2008; Bouzat et al., 2013; Chen et al., 2015; Logue et al., 2015). While autochthonous sources of OM are preferentially degraded and readily mineralized into CH_4_ (West et al., 2012, 2015), the vast majority (87%; Kritzberg et al., 2004) of dissolved organic C (DOC) in lakes is from terrestrial sources, and is considered more recalcitrant (Kritzberg et al., 2004). Recalcitrance has partly been linked to increasing abundance of polyphenolic compounds, which have been shown to inhibit extracellular bacterial enzymes involved in decomposition, and have a toxic effect on methanogens (Field et al., 1988; Wetzel, 1992; Ye et al., 2016), but little literature exists exploring the effects of OM inputs from shifting plant communities on bacteria or methanogens in lake sediments. An even larger gap exists when it comes to exploring fungi in lake sediments, as research has largely focused on the syntrophy of bacteria and methanogens (e.g., Bastviken et al., 2008; Mcinerney et al., 2009). Despite some evidence that fungi are important decomposers in other anaerobic systems (e.g., Brad et al., 2008), little effort has been given to examine their role in lake sediments (Wurzbacher et al., 2010).

Therefore, widespread and rapid changes in plant community composition in catchments of freshwater systems—resulting from different land use practices and/or climate change (Foley et al., 2005; Millar et al., 2007)—have the potential to shift lake sediment bacterial, fungal, and methanogen communities, and subsequently impact lake-level C emissions. Previously, it has been shown that deforestation can reduced DOC concentrations (g/m of shoreline) by 98% (France et al., 1996), which emphasizes how abrupt changes in plant communities around lakes can have sudden and large impacts.

Here we asked how shifting plant communities and litter types influence microbial communities in lake sediments. Previously, we found that the decomposition of plant litter from the emergent macrophyte *Typha latifolia* released at least 400-times more CH_4_ than that of terrestrial plant litters (Emilson et al., 2018). *T. latifolia* loading in sediments also increased methanogen abundance in contrast to forest litters (Emilson et al., 2018). Furthermore, an analysis of the DOM components revealed much higher polyphenol levels in the forest litters than in the *T. latifolia*, which appeared to suppress methanogenesis and methanogen cell propagation (Emilson et al., 2018). Given that climate change is expected to increase emergent macrophyte coverage in temperate and boreal lakes (Jeppesen et al., 2009; Alahuhta et al., 2011), these results, along with the other literature cited, warrant further examination of the specific changes to microbial communities. Here we use the same samples from Emilson et al. (2018) to ask: (1) Do bacterial, fungal, and methanogen communities vary in composition and richness with different plant litters? (2) Does pore-water DOM characteristics—particularly polyphenol content—during plant litter decomposition influence dominant bacterial, fungal or methanogen taxa? (3) What are the fungal communities associated with anaerobic decomposition of plant litters in lake sediments? (4) What is the role of the native microbial community structure, e.g., as seen between lakes with high vs. low sediment OM, in processing new litter inputs? To address these questions, we used a microcosm approach, and analyzed lake sediment microbial community responses to amendment with different plant litters.

## Materials and Methods

### Experimental Design

Sediments for anaerobic incubations were constructed by amending low OM content (0.3% dry weight) lake sediments collected from a single location, consisting of the top 5 cm of sediments [where a majority of methanogenesis occurs in sediments (Van Den Pol-van Dasselaar and Oenema, 1999)] from Geneva Lake, Ontario, Canada (46°45′27.2″N, 81°33′19.8″W). Collected sediments were homogenized to ensure similar starting conditions prior to amendment. Amendments consisted of a boreal species coniferous needle mix (CON; *Pinus resinosa* and *P. strobus*), deciduous leaf mix (DEC; *Acer rubrum*, *A. saccharum*, *Betula* spp., *Populus tremuloides*, *Ulmus americanum*, *Quercus rubra*, and *Q. alba*), and the emergent macrophyte *Typha latifolia* (TYP)—amended treatments are hereafter referred to by their respective abbreviations. The leaf litter was first oven dried for 12 h at 60°C, then ground and sieved to obtain and utilize only the fine particulate size fraction ( ≤ 1 mm). The raw leaf litter mixes were processed alongside the amended sediments, and hereafter are referred to as leaf litter controls. Each organic matter type was incorporated into the natural lake sediment at either a 10, 20, or 40% dry weight concentration. Our amendment concentrations were chosen to enrich for sediment microbial communities that specialize in the decomposition of the respective litters that can occur due to rapid changes in riparian and catchment vegetation. Sediments were placed in 250 mL mason jars to create a vertical depth of 4.5 cm to allow for expansions with wetting to a total simulated depth of 5 cm, then saturated with TOC-scrubbed A10 MilliQ water (EMD Millipore Corp., Darmstadt, Germany). Along with amended sediments, unamended sediments were also placed into jars and incubated, and hereafter referred to as CTR (incubated sediments). Each treatment was created in quadruplicate. The jars were then sealed with a lid fitted with a rubber septa, and headspace air was removed and replaced with N_2_ to create anaerobic conditions.

**FIGURE 1:**
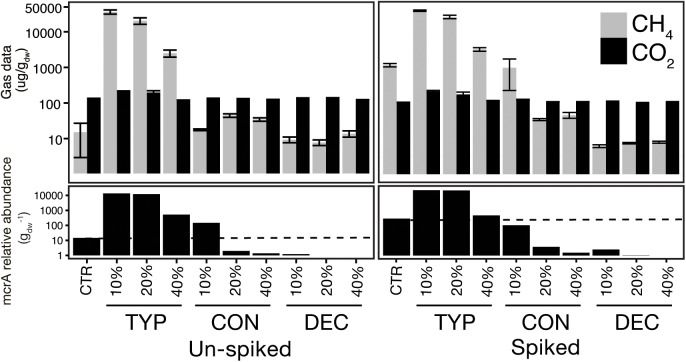
Total CO_2_ and CH_4_ produced shows the modulating effects of plant litter on methanogenesis and methanogen abundance. The top panel shows mean cumulative production of CH_4_ and CO_2_ over the 150-day incubation for each plant litter treatment, across each percent organic matter addition, with error bars indicating ± SE (*n* = 4). The bottom panel shows the corresponding *mcrA* copy number across treatments, set relative to the un-spiked control, with the dashed lines indicating the respective control copy number.

We constructed a second set of the same amendments as described above but with a “spike” of 5% by dry weight of sediments from Ramsey Lake, ON (46°28′19.8″N, 80°58′19.2″W) which contained 3% by dry weight OM. The sediment spike was used as a microbial inoculum without adding much additional OM. The spike sediments were obtained from an area of Ramsey lake known to be highly methanogenic relative to Geneva Lake, and spiked sediment controls (unincubated sediments) were found to have higher copy numbers of the *mcr*A gene as well as greater CH_4_ production (Figure 1). OM content of each sediment was obtained by oven drying subsamples at 60°C to get a dry weight conversion, and subsequently combusted at 550°C for 4 h to calculate OM%. All sediments were incubated at 20.5°C for 150 days. During trial experiments of our setup, the maintenance of anoxic conditions was confirmed throughout incubation periods via Oxoid Anaerobic Indicator strips (Thermo Fisher Scientific, Waltham, MA, United States).

At the end of the incubation, we characterized microbial communities by extracting sediment DNA, and related community composition and abundance to physicochemical data, namely CO_2_, CH_4_, and DOM fluorescent measurements (for more details see Emilson et al., 2018). Physicochemical data were collected for each of the four replicates of each treatment, and included periodic measurements of CO_2_ and CH_4_ concentrations by gas chromatography (SRI 8610C with a 0.5 mL sample loop and 105°C), and the total concentration and chemical characteristics of dissolved organic matter at the end of the experiment. As many anaerobic decompositional processes are acid generating, we measured post-incubation pore water pH using a HI 9126 pH meter (Hanna Instruments, Woonsocket, RI, United States). Dissolved organic matter fluorescence excitation-emission matrices (EEMs) were generated from the pore water of each sample using an Agilent Cary Eclipse Fluorescence Spectrophotometer in ratio (S/R) mode with a 1-cm path length cuvette, with EEMs generated from excitation and emission intensities. EEMs were adjusted for inner-filter effects by absorbance with measurements from an Agilent Cary 60 UV-Vis Spectrophotometer. A total of five components were identified via PARAFRAC modeling (referred to as C1–C5 defined by maximum excitation/emission intensities). Briefly there were humic-like components C1 (310/414 nm) and C2 (345/462 nm), a tryptophan-like protein component C3 (280/354 nm), a tyrosine-like protein components C4 (270/306 nm), and to a protein-like polyphenol component C5 (275/318 nm) (Maie et al., 2007, 2008; Yamashita et al., 2008).

### DNA Extractions

On day 150, sediments in each jar were homogenized, and then sub-samples were taken, immediately frozen and then stored at -20°C for microbial community analysis. Each treatment quadruplicate sample was thawed, and DNA was extracted in duplicate using the MoBio PowerSoil kit (MoBio, Carlsbad, CA, United States). Quadruplicates from each treatment were paired, and had their DNA extracts combined, resulting in duplicate DNA samples representing each treatment, i.e., four replicates of the TYP 10% was reduced to two for sequencing (each constituting four DNA extractions from two jars). Pooling DNA was done to reduce bias of small material amounts that makes up one DNA extraction, and to increase DNA concentrations for downstream work. DNA was concentrated down into 40 μL aliquots suspended in the C6 solution from the MoBio PowerSoil kit. Sample DNA was quantified using a Take3 spectrophotometry system on a Synergy HI microplate reader (BioTek, Winooski, VT, United States).

### Amplicon Sequencing

Sequencing of duplicate DNA samples representing each treatment was carried out on an Illumina MiSeq system (Illumina Biotechnology Co., San Diego, CA, United States) by Metagenome Bio Inc. (Toronto, Canada), with three targets: general prokaryote primers to target bacterial 16S rRNA genes Pro341F (5′-CCT ACG GGN BGC ASC AG-3′) and Pro805R (5′-GAC TAC NVG GGT ATC TAA TCC-3) (Takahashi et al., 2014); *mcrA* primers to target methanogens mlas (5′-TGG YGG TGG TMG GDT TCA CMC ART A-3′) and mcrA R (5′-CGT TCA TBG CGT AGT TVG GRT AGT-3) (Angel et al., 2011); and ITS primers to target fungi ITS1F (5′-ACC TGC GGA RGG ATC A-3′) and ITS1R (5′-GAG ATC CRT TGY TRA AAG TT-3′) (Bokulich and Mills, 2013). Primers had index sequence (6 bp) overhangs added to facilitate sample pooling prior to sequencing. A 25 μl PCR reaction was performed containing 5 μl of standard OneTaq buffer (5x), 0.25 μl of 25 mM dNTP, 0.5 μl of both the forward and reverse primers (10 μM each), 1 μl BSA (12 mg/ml), 0.125 μl of OneTaq DNA polymerase (5000 units/mL, New England Bio, MA, United States), 1–10 ng DNA and water to equal a total reaction volume of 25 μl. Initial denaturation started at 94°C for 5 min followed by 30 cycles of 94°C for 30 s denaturation, annealing was at 53°C for 16S, 62°C for both 18S and *mcr*A for 45 s, extension at 68°C for 1 min with a final extension step at 68°C for 10 min. PCRs were done in triplicates for each sample to reduce PCR bias, and products were checked on a 2% agarose gel, after which the bands were excised with a MinElute gel extraction kit (Qiagen, Hilden, Germany). The purified DNA libraries were quantified on a Qubit with the dsDNA HS assay kit (Life Technologies, CA, United States), the library pools were spiked with 5% phix control (V3, Illumina) to improve base imbalance, and paired-end sequencing with read lengths of 250 bp was performed using MiSeq Reagent kit V2 (2 × 250 cycles) on a Illumina MiSeq platform.

### Quantitative PCR (qPCR)

As the primary differences in CH_4_ production and microbial communities were found to be between litter types, and not concentrations of litter, DNA for each litter type was further pooled for quantitative PCR (qPCR). qPCR was carried out with technical triplicates to determine relative abundance of bacteria, fungi, and methanogens. qPCR has been shown to be a reliable metric to compare relative abundance of fungi and bacteria, and generally mirrors biomass dynamics (Fierer et al., 2005; Rousk et al., 2010). The *mcr*A qPCR data were first presented in Emilson et al. (2018) the methodologies are re-iterated here for the convenience of the reader. Primers targeted: bacterial 16S rRNA gene using Eub-338 (5′- GCT GCC TCC CGT AGG AGT-3′) and Eub 518 (5′- ATT ACC GCG GCT GCT GG-3′) (Fierer et al., 2005); Fungal 18S rRNA gene using FU18S1 (5′-GGA AAC TCA CCA GGT CCA GA-3′) and Nu-SSU-1536 (5′-ATT GCA ATG CYC TAT CCC CA-3′) (Gangneux et al., 2011) while we used the same primers for detecting methanogens as for the Illumina *mcr*A sequencing. The qPCR conditions (16S/*mcr*A/18S genes, respectively) were a 5-min initial denaturation at 95°C, followed by 30/35 cycles of 95°C for 10 s, 53/55/62°C for 10 s, and 72°C for 10 s with a final denaturation for 1 min at 95°C. The qPCR was done using Bio-Rad’s iTaq universal Sybr green Supermix on an Agilent Technologies Stratagene Mx3005P. Copy numbers for bacteria and fungi were calculated using standard curves of *Escherichia coli* (ATCC#11303) and *Saccharomyces cerevisiae* (ATCC#2360), respectively, giving final units of copies per gram of dry weight of sediment. A standard curve was made for *mcr*A using a serially diluted purified PCR product of amplified DNA, which was run in triplicate, and values were standardized relative to the control and per gram of dry weight. All qPCR runs had *R*^2^ values greater than 99% and efficiency values greater than 90%. Final product purity was confirmed via dissociation curve and on a 1.5% agarose gel (Supplementary Figure S1).

### Data Analysis

An average of 43 799 (SD 16 375) 16S rDNA reads, 28 761 (SD 14 825) ITS reads, and 14 267 (SD 12 176) *mcr*A reads per sample after quality filtering were obtained during sequencing on the MiSeq. Raw Miseq reads were merged using PANDAseq for the *mcr*A and 16S rRNA gene reads, and using PEAR for the fungal ITS fragments (Masella et al., 2012; Stamatakis et al., 2014). All reads were then further quality filtered by removing chimeras and OTUs were assigned using USEARCH v8.1.1861 at a 97% similarity threshold for 16S and ITS reads, whereas 85% was used for *mcr*A reads according to the specification of Yang et al. (2014). Taxonomy was assigned through QIIME using the RDP classifier (Wang et al., 2007) for the 16S rRNA gene reads and *mcr*A reads, with the Green Genes database (DeSantis et al., 2006) and a database created by Yang et al. (2014), respectively. For the ITS fungal reads the BLAST (Altschul et al., 1990) aligner was used with the UNITE database (Koljalg et al., 2013). Data were then imported and analyzed in R using the phyloseq package (McMurdie and Holmes, 2013; R Development Core Team, 2016). Abundance data from sequencing were first normalized using the DeSEq2 package in R (Love et al., 2014). The sequencing data have been deposited into NCBI under BioProject PRJNA347436, containing SRA samples SRR4418117-SRR4418160.

Phylogenetic trees were constructed for UniFrac (distance matrices that incorporate phylogenetic distances) analysis for each the bacteria, fungal, and methanogen data sets. An analysis was also done using subset OTUs with relative abundance >0.01% to test if the rare biosphere impacts overall community structure. Sequences were first aligned using MUSCLE, and the resulting alignments manually trimmed in MEGA7 (Edgar, 2004; Kumar et al., 2016). Maximum likelihood trees were then constructed using RAxML, with 1000 bootstrap replicates (Stamatakis, 2014). To further examine microbial communities, the Chao 1 richness estimate was calculated with the Fossil package (Vavrek, 2011) in R using normalized read counts with singletons and doubletons removed to reduce biases in richness estimates by the abundance of rare OTUs inherent in high-throughput sequencing.

Gas, pH, qPCR, DOM data and relative abundance of OTUs determined from sequence libraries were analyzed for significant relationships using Pearson’s product moment correlations, one- and two-way analysis of variance (ANOVA) where appropriate, and a Permutational Multivariate Analysis of Variance using Distance Matrices (ADONIS) using core R and the vegan package (R Development Core Team, 2016; Oksanen et al., 2017). Indicator species analyses were performed using the Indicspecies multipatt function in R (De Caceres and Legendre, 2009) and the gplots Heatmap.2 function (Warnes et al., 2016). UniFrac distances, both unweighted (presence absence of OTUs) and weighted (uses relative abundances of OTUs) and PCoA plot construction was done using the Phyloseq R package (McMurdie and Holmes, 2013). To analyze the top most abundant bacterial taxa, sample relative abundances were plotted across samples from DEC (containing the highest measured polyphenols) then sequentially CON, and TYP (containing lower polyphenol levels) and ordering each plant litter’s samples from 40 to 10% of both un-spiked and spiked treatments. This allowed the variation in dominant bacterial taxa at various taxonomic levels to be visualized across a pseudo-polyphenol concentration gradient.

## Results

**FIGURE 2:**
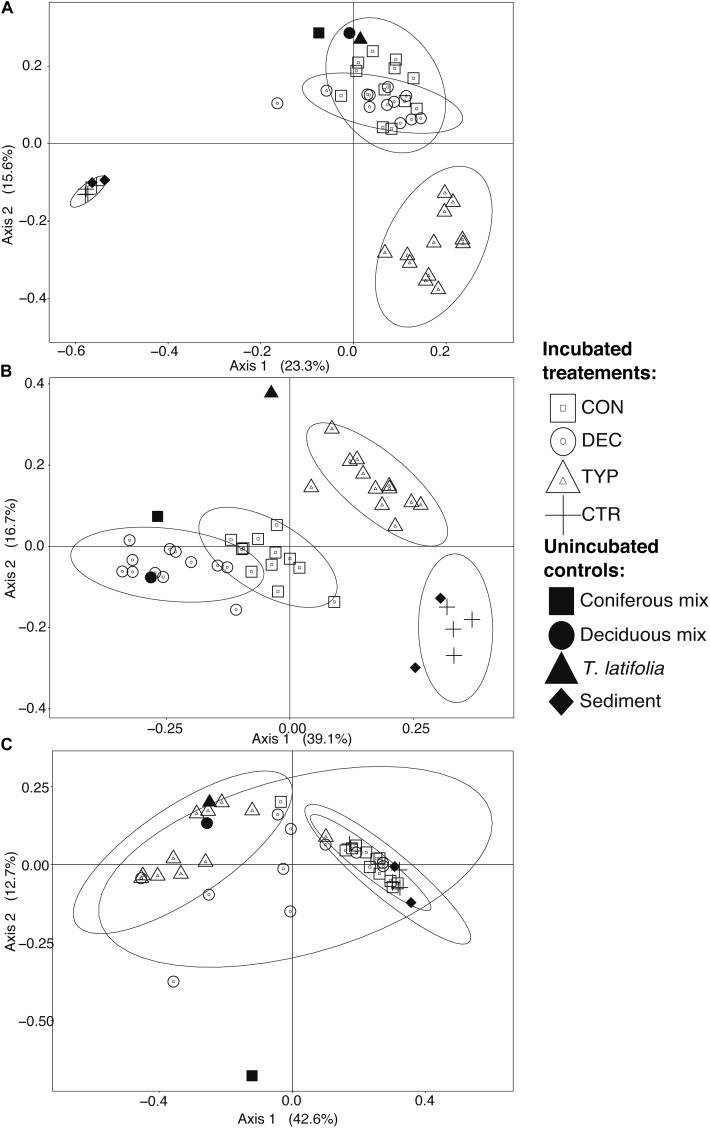
PCoAs of un-weighted UniFrac distances using relative abundances for the respective microbial communities from both un-spiked and spiked samples; bacteria (**A**; 5565 OTUs), fungi (**B**; subset of 131 OTUs), and methanogens (**C**; 43 OTUs), across all treatment concentrations. Ellipses represent 95% confidence intervals which were calculated for each incubated treatment. The proportion of variation explained by each axis is given in parentheses. The pre-incubated sediment is both the un-spiked and spiked sediments.

We found that microbial communities varied more among litter types than concentrations and were similar between spiked and un-spiked sediments (Figure 2). For these reasons, we grouped all samples by litter type to visualize differences in Figure 2. Generally, litter type explained more variation than organic matter concentration (ADONIS test for litter type and concentration, respectively: bacteria: *R*^2^ = 0.52, *p* < 0.001 and *R*^2^ = 0.04, *p* < 0.001; methanogens: methanogens *R*^2^ = 0.42, *p* < 0.001 and *R*^2^ = 0.04, *p* = 0.041). Whereas, fungal communities only varied significantly across litter types (ADONIS test for litter type and concentration, respectively: *R*^2^ = 0.86, *p* < 0.001, and *p* = 0.242). PCoA ordination of unweighted UniFrac distance matrices further revealed that bacterial and fungal communities in the CON and DEC samples were different from those in the CTR and TYP amendments (Figures 2A,B). The methanogen communities showed less heterogeneity in the both the CON and TYP treatments compared to the DEC (Figure 2C). Communities in all sediment controls remained similar to the starting sediments after 150 days of incubation (Figure 2).

### Community Structure and Richness

**FIGURE 3:**
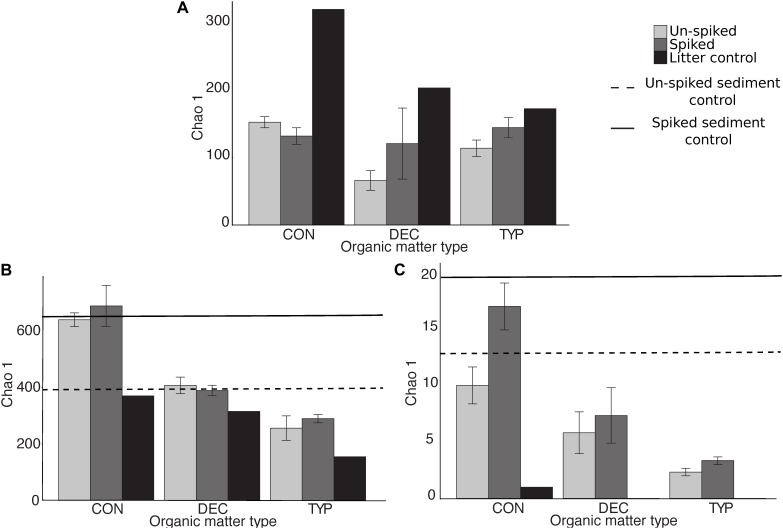
Mean diversity (±SE) of bacterial **(A)**, fungal **(B)**, and methanogen **(C)** communities for each plant litter type. Diversity was calculated as Chao 1 values averaged for each plant litter type added to either the un-spiked or spiked amended sediments and pre-incubation plant litter controls. Bacterial values for both the un-spiked and sediment control were 975.5 (±77.5) and 1360.5 (±111.5), respectively, and beyond the *y*-axis limits therefore not included in the figure. SE for fungal un-spiked sediment control was ±6 and ±171 for the spiked, and ±3.5 and ±0.5, respectively, for methanogens.

The different plant litters resulted in differing communities, however, more similarities existed between CON and DEC communities than either with the TYP communities (Figure 2A; unweighted UniFrac). When bacterial relative abundances were considered, TYP and CON treatments were more similar (Supplementary Figure S2; weighted UniFrac). In all cases, the plant litter amendments resulted in lower bacterial Chao 1 values by 17–59% compared to the litter controls (the starting litter material), and 84–95% decrease compared to the sediment controls (the starting sediments: Figure 3A). Even with this reduction in bacterial richness across plant litter treatments, we did not observe any distinct indicator OTUs for specific plant litter types.

**FIGURE 4:**
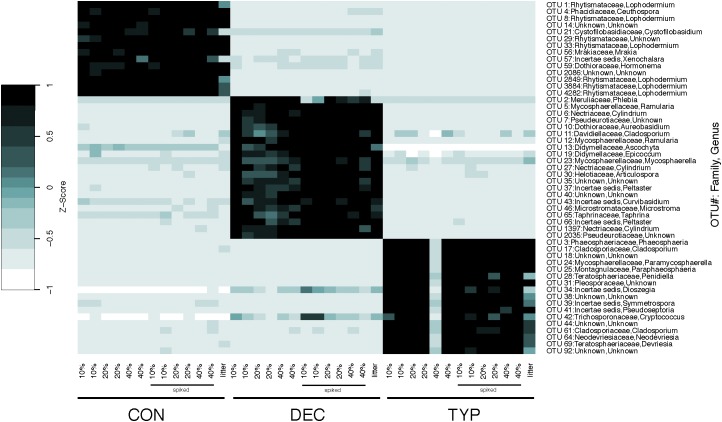
Heat map of indicator fungal OTUs for replicate DNA extractions by organic matter type and their respective starting litter material. OTU number and assigned taxonomic information are identified on the right *y*-axis, with the lowest identifiable taxonomic rank in brackets when Family and Genus were unknown. The leaf litter control for each material is shown last on the *x*-axis for each litter type.

The fungal communities were largely unique across plant litter types, with the different litters resulting in different compositions compared to those in the sediment and leaf litter controls, except for the DEC treatment (Figure 2B). Although there was a slight overlap of the confidence intervals between the CON and DEC, differences between the communities were strongly dictated by litter type (Figure 2B). Plant litter amendments also resulted in an increase in fungal richness, reflected in Chao 1 values increasing by 23–86% relative to the litter controls (Figure 3B). However, an increase in fungal community richness was observed in the CON treatment relative to both the un-spiked and spiked sediment controls by 62 and 6%, respectively, but was relatively similar or decreased between 0 and 55% in the DEC and TYP treatments in comparison to the spiked sediment. The starting sediment fungal communities contained Ascomycota sequences, as well as some Basidiomycota, Rozellomycota (also classified as Cryptomycota), and some Glomeromycota in the spiked sediments (Supplementary Figure S3). After litter amendment, specific OTUs dominated the different litter types. From the communities for each litter type a total of 52 indicator OTUs were identified, with a majority identifiable at the family and genus levels (Figure 4). These indicators were part of the subset of the 131 of 4196 most abundant fungal OTUs (>1% within at least one sample).

**FIGURE 5:**
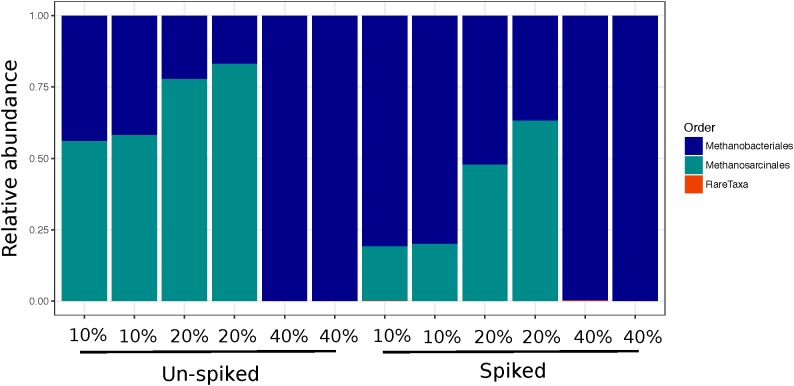
Relative abundance bar plots of *mcr*A sequences showing methanogen orders in the TYP samples for each sequencing duplicate at each concentration for both the un-spiked and spiked treatments.

Across all plant litters, we found an increase in methanogen Chao 1 relative to leaf litter controls (Figure 3C), with both DEC and TYP plant litters having no detectable methanogen OTUs, and CON having only 1. Overall, Chao 1 values for the methanogens increased between 3 and 17% in the litter amended sediments relative to the litter controls. However, richness decreased after incubation relative to both the un-spiked (2.5–10%) and spiked (13–83%) sediment controls. The DEC and CON methanogen communities both had low *mcr*A copy numbers, with several OTUs unidentifiable at the order level with some Methanobacteriales, Methanosarcinales, and *Methanomassiliicoccus* (see Supplementary Figure S4 for a phylogenetic tree). The TYP 10 and 20% OM samples were primarily composed of two orders of methanogens, Methanosarcinales (OTU 2) and Methanobacteriales (OTU 1), whereas both un-spiked and spiked TYP 40% OM samples were dominated by Methanobacteriales (Figure 5). This shift in the dominant methanogen taxa was linked to the total amount of CH_4_, as members of the methanogen family Methanosarcinaceae were the only group that were positively correlated with total CH_4_ production across 150 days (*r* = 0.49, *p* < 0.005). Additionally, the TYP treatments started producing CH_4_ in order of increasing OM concentration (10% started producing on day 31, 20% on day 60 and 40% on day 91), with 10% having produced the most by day 150, then the 20 and 40% treatments (Supplementary Figure S5).

### Microbial Associations With DOM and pH

**FIGURE 6:**
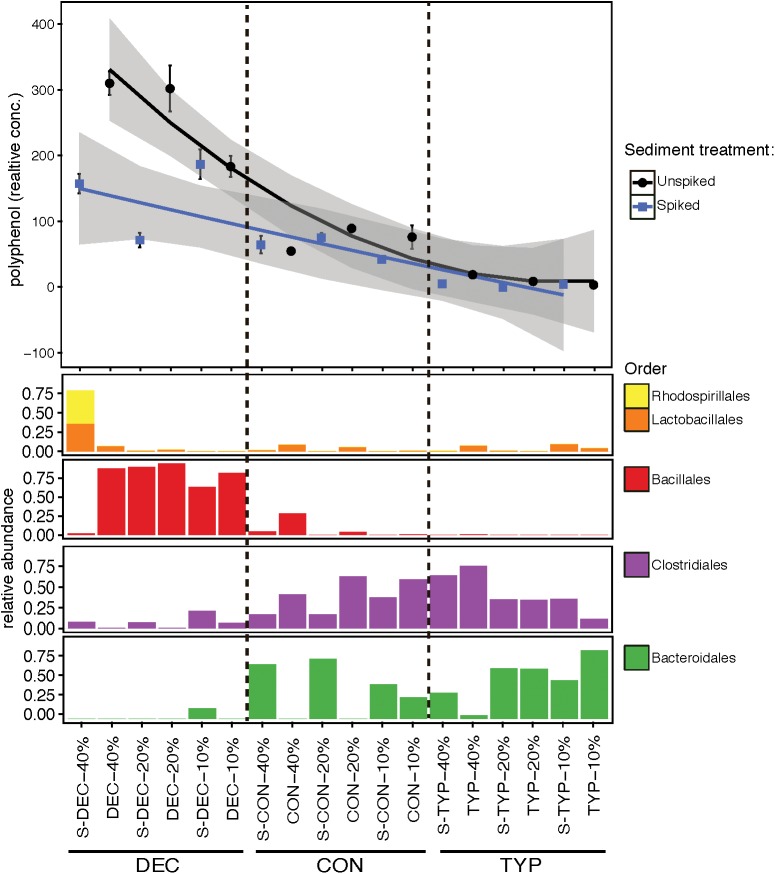
Relative polyphenol concentrations across leaf litter types and dominant bacterial orders. Polyphenol concentrations were standardized to relative concentrations of total dissolved organic carbon. Spiked samples are indicated on the *x*-axis labels with S. The *x*-axis was ordered to create a pseudo-concentration gradient of polyphenols across three plant litters with dashed lines denoting different litter types. Polyphenol levels were averaged for each treatment and bars represent standard errors with the shaded area around each trend line representing the 95% confidence interval. Relative abundances from sequencing duplicates were averaged for every OTU to represent each treatment and were then summed by Order.

Variation in the bacterial communities was related to relative polyphenol concentration across amendments (Figure 6), in contrast to the same starting sediment communities. In the starting sediment controls the bacterial community was similar between the spiked and un-spiked samples, containing primarily sequences from Proteobacteria, Nitrospirae, Chloroflexi, and the spiked-sediment control also contained Bacteroidetes and Cyanobacteria (Supplementary Figure S3). The un-spiked and spiked treatments were mostly similar across litter amendments except for between the CON 20 and 40% OM treatments, where Bacteroidales (Bacteroidetes) were only found in the spiked samples. An additional difference was between the highest polyphenol containing samples of DEC litter with a 40% OM concentration. The spiked DEC 40% OM treatment was dominated by Rhodospirillales (comprised of *Acetobacter* spp. OTUs; Alphaproteobacteria), and Lactobacillales [largely comprised of *Leuconostoc* spp. (Firmicutes) and OTUs unidentifiable at the genus level, Supplementary Figure S6]. The un-spiked DEC 40% OM along with the other DEC concentrations, both spiked and un-spiked, were dominated by OTUs from the order Bacillales (primarily OTU 1, which is a *Sporolactobacillus* sp.; Firmicutes). Lower abundance bacterial orders included Enterobacteriales (Gammaproteobacteria), Coriobacteriales (Actinobacteria), and OPB54, none of which showed clear patterns across treatments (Supplementary Figure S6). Differences in pH were also observed between the spiked DEC (mean = 3.62; *SE* ±0.25) and un-spiked (mean = 4.20; *SE* ±0.1) treatments, and DEC had a lower pH than either the CON or TYP treatments with both un-spiked and spiked sediments (two-way ANOVA: *F*_3,72_ = 7.99, *p* < 0.001, Tukey’s: *p* < 0.001 for all comparisons). The differences in pH were also reflected in the sediment controls, with the un-spiked control having a mean pH of 6.94 (*SE* ±0.24), and the spiked being 4.93 (*SE* ±0.32). All mean pH values are presented in Supplementary Table S1.

Bacterial 16S rDNA and methanogen *mcr*A abundances were linked to DOM characteristics and in turn to CO_2_ production. Our analysis of DOM characteristics revealed that bacterial and methanogen abundances positively correlated with humic-like components C1 (16S rDNA *r* = 0.68, *p* = 0.002; *mcr*A *r* = 0.72, *p* < 0.001), and C2 (16S rDNA *r* = 0.68, *p* = 0.002; *mcr*A *r* = 0.69, *p* = 0.002), whereas the C4 protein-like component correlated with just bacterial abundance (16S rDNA *r* = 0.49, *p* = 0.047). Increases in bacterial and methanogen abundances also corresponded with an increase in CO_2_ production (respectively: *r* = 0.56, *p* = 0.021; *r* = 0.96, *p* < 0.001), and bacterial abundance were associated with TYP amendment (one-way ANOVA: *F*_5,16_ = 25.6, *p* < 0.001; Tukey’s test for all comparisons including TYP: *p* < 0.001). In general, these patterns revealed how TYP enhanced both bacterial and methanogen activity as well as cell propagation.

### Fungi vs. Bacteria

The ratio of fungi to bacteria was examined to determine how different plant litters affected the dominant decomposers. We found a negative correlation between bacteria and fungi (*r* = -0.60, *p* = 0.010: qPCR values summarized in Table 1). Further examination revealed that bacterial 16S rDNA copy number and polyphenols negatively correlated (*r* = -0.73, *p* < 0.001) while fungal 18S copies positively correlated with polyphenol levels (*r* = 0.83, *p* < 0.001). Polyphenol concentrations were different amongst plant litters, and were highest in DEC samples (one-way ANOVA, *F*_2,14_ = 15.66, *p* < 0.001; Tukey’s, DEC-CON *p* = 0.0146, DEC-TYP *p* = 0.001), which corresponded to a higher copy number of fungi (one-way ANOVA, *F*_2,14_ = 16.62, *p* < 0.001; Tukey’s, DEC-CON *p* = 0.004, DEC-TYP *p* < 0.001). Examining the increase in fungal abundance further, we observed a positive correlation with *Phlebia* spp. (*r* = 0.79, *p* < 0.001), which had high relative abundances in the DEC amended sediments. On average the relative abundance of *Phlebia* spp. ranged between 5 and 40% in DEC samples, whereas it was below 0.01% for all other treatments and controls. These results demonstrated a dominance of fungi over bacteria in the presence of high polyphenol containing litter, even in an anaerobic environment.

**Table 1:** Quantitative PCR of Bacteria (16S) and Fungi (18S) in sediments and the starting litter OM used to amend the treated sediments.

Treatment	*N*	Fungal^1^ copies	Bacterial copies	Ratio of fungi to bacteria	Relative^2^ polyphenol levels (%)
CON	5	3.63 × 10^4^ (1.29 × 10^4^)	2.77 × 10^5^ (4.93 × 10^4^)	0.13 (0.029)	21 (0.015)
DEC	6	9.51 × 10^4^ (1.34 × 10^4^)	6.87 × 10^4^ (1.89 × 10^4^)	1.9 (0.518)	43 (0.075)
TYP	6	1.34 × 10^4^ (2.59 × 10^4^)	9.63 × 10^5^ (1.13 × 10^4^)	0.013 (0.001)	6.6 (0.016)
CON Litter control	1	5.32 × 10^6^	3.63 × 10^4^	146.3	37 (0.18)
DEC Litter control	1	1.89 × 10^6^	7.53 × 10^4^	25.2	34 (1.13)
TYP Litter control	1	4.24 × 10^6^	2.58 × 10^5^	16.5	12 (1.67)


*^1^Gene copy number was standardized per g/dry weight.*

*^2^N for litter polyphenol leachate measurements is 4.*

*Values are the mean copy number of spiked and un-spiked samples with standard error in brackets calculated from standard curves of pure cultures. Total N for the experimental treatments included all amendment concentrations*.

## Discussion

Here we examined the lake sediment microbial communities involved in decomposing different types of plant litters. We found that community composition and richness changed in response to biochemical differences created by different plant litters, which had an environmental filtering effect on the microbial communities, changing either composition and/or abundance of the respective communities. While we did not directly observe interactions between different microbial taxa, we can infer that the changes in the initial decomposer resulted in differing methanogen communities due to differences in metabolic capabilities and therefore by-products. Future studies could survey the changes in metabolic genes being transcribed during different stages of decomposition and test how they vary across different litter types. Such an analysis would reveal how changes in community composition alters the flow of carbon metabolites through the system (e.g., see Woodcroft et al., 2018). Additionally, exploring how fluctuations in temperature could affect the respiration rates and ratios of sediment fungi and bacteria (e.g., Pietikainen et al., 2005) would provide insights into longer term dynamics over seasons. These findings extend the conclusions of previous work linking microbial community dynamics in lake waters to DOM sources (Logue et al., 2015). Our results agree with past literature that emphasizes the importance of the chemical composition of plant litter when evaluating decomposition rates, not simply litter species richness (Wardle et al., 1997; Lecerf et al., 2007; Schindler and Gessner, 2009; Swan et al., 2009). We extend this paradigm to lake sediments, and find fungi as well as bacteria and methanogens as important decomposers in anaerobic lake sediments.

Our results here along with past studies (e.g., Hoostal and Bouzat, 2008) demonstrate that natural lake sediments range in their abilities to process fresh leaf litter, and this in part is modulated by polyphenolics from terrestrial plant litters. In our current study, polyphenols had strong correlations with bacterial, fungal and methanogen community structure, and abundances of bacteria and fungi. While the DEC and CON starting litters leached a similar proportion of polyphenols into the pore water, post-incubation the DEC treatments maintained a higher relative abundance of polyphenols (Table 1). It has been shown previously that deciduous tree leaf litter generally leaches more polyphenols than coniferous (Kuiters and Sarink, 1986), which is consistent with our endpoint pattern (Figure 6).

Our results also indicate that the initial microbial inoculum of lake sediments can also affect how OM is decomposed. In the spiked DEC treatment, polyphenol levels were lower than the un-spiked DEC treatment (Figure 6), suggesting the spike of high OM sediments contained microbes able to metabolize polyphenols that were absent in the un-spiked community. Differences in the metabolic capabilities of the spiked communities were also reflected in the lower pH values, suggesting increased acid generation. However, our current study design does not provide the functional data necessary to fully elucidate the differences in the biochemical processing of the polyphenols between treatments, or the relationships between the humic components (C1 and C2)—which could potentially contain polyphenolic compounds—and the protein-like polyphenolic component (C5). However, our results suggest that polyphenols play an important role in shaping sediment bacterial communities, and this warrants future study using techniques to examine the DOM fractions in higher resolution. Adapting to high polyphenol concentrations could be important to C cycling in lake sediments, as polyphenols are often regarded as inhibitors of decomposition and nutrient cycling (Hättenschwiler and Vitousek, 2000).

### Bacterial Communities

We observed a reduction in bacterial richness with increasing concentrations of plant litter, and hypothesize that this reflects a specialization of the community (Figure 3A). A similar trend was previously observed using additions of OM, and is surmised to be the effect of environmental filtering, as the bacteria that dominate are adapted to thrive in the chemical environments created by different types of OM (e.g., Sato et al., 2016). Comparing the un-weighted UniFrac distances (which incorporate presence-absence data with phylogenetic distances; Figure 2A) with the weighted UniFrac distances (which also incorporated relative abundances) (Supplementary Figure S2A) revealed the selection of certain bacterial taxa across increasing polyphenol levels across litter types (pattern displayed in Figure 6).

Bacterial abundances were positively associated with increased CO_2_ production, indicating they were the drivers of a more active decomposition versus fungi (fungi discussed more below). We also saw a strong correlation of methanogen abundances to CO_2_ production, which is likely due to their reliance on bacterial syntrophs to produce necessary precursors for methanogenesis. Therefore, in our short-term experiment, the TYP and CON amended sediments communities could mineralize more carbon (Figure 1). Multi-year experiments would need to be performed to analyze whether the carbon in the DEC samples could be mobilized by microbial activity, and if any eventual methanogenesis could occur.

### Fungal Communities

Differences in abundances of bacteria and fungi revealed a certain degree of niche partitioning, with fungi showing greater abundance in the DEC treatments than bacteria. A *Phlebia* spp. was identified as a key OTU for DEC litters with a strong positive relationship to polyphenols. *Phlebia* are often lignin degrading white rot fungi, which have been shown to have high polyphenol oxidase activity (Talbot et al., 2015). However, our data suggested that metabolic activity decreased in the DEC samples, with lower overall CO_2_ production (Figure 1). This could be due to lower growth rates and metabolic rates of the fungi in the anaerobic conditions. The lack of methanogenesis in the DEC samples also indicate the fungi present are unable to produce the same by-products (like acetate) that bacteria do, which are used by methanogens to generate energy and methane. We would predict that if the incubations were run longer, as the fungi decomposed the polyphenols in the samples, more bacteria would be able to grow, and start more rapidly decomposing the plant litter, resulting in increases in both CO_2_ and CH_4_. Although symbioses of bacterial and fungal decomposers has been explored in terrestrial systems (e.g., De Boer et al., 2005), to our knowledge no one has explored their dynamics in lakes sediments. Data presented here suggests that the dynamics of fungal and bacterial decomposers could be important to understanding decomposition in lake systems, which is an important future avenue of research as the phenolic compound levels in plant tissues is predicted to increase with rising atmospheric CO_2_ levels (Tuchman et al., 2002).

The common paradigm that bacteria dominate anaerobic sediments and soils over fungi has led to fungi being often ignored or not reported in literature examining decomposition of OM in lake sediments. In aerobic conditions, plant litter is colonized by fungi that often have greater biomass than bacteria (e.g., Newell et al., 1995; Findlay et al., 2016). The aerobic activities of fungi as decomposers contributing to C cycling in aquatic systems has been observed and acknowledged (see Bärlocher, 2016), however, little prior evidence suggest fungi play roles in anaerobic degradation of organic inputs to sediment. With the presence of high polyphenols in the DEC treatment samples it is likely that bacterial decomposers were out-competed by fungal taxa better adapted to metabolizing polyphenols (i.e., *Phlebia* spp.). It is also likely the litters leached polyphenols with anti-microbial properties that contributed to an environmental filtering effect, as different polyphenolic compounds can be inhibitory to different microbial groups (Daglia, 2012). The opposite case was likely true with the lower polyphenol conditions, where the fungi were unable to compete as well with bacteria.

### Methanogen Communities

Methanogens were the only functional microbial group examined here in detail (specifically sequenced and quantified via qPCR), and were found to be seemingly un-important in the forest litter decomposition with low methanogenesis and cell numbers, but the opposite was true for the TYP treatments. Over the 150-day incubation we observed increasing amounts of CH_4_ production from amended sediments, and sequencing revealed a specific taxon responsible for carrying out methanogenesis. Overall the TYP treatment communities were dominated by Methanosarcinaceae (OTU 2, grouping with *Methanosarcina barkeri* in Figure 5), and Methanobacteriaceae (OTU 1, further identified as *Methanobacterium* sp. swan-1). Increasing CH_4_ production correlated with the increase in Methanosarcinaceae in the 10 and 20% OM TYP treatments, and notably members of this family have relatively wide substrate utilization capabilities (Liu and Whitman, 2008; De Vrieze et al., 2012).

In contrast the 40% OM treatment (both un-spiked and spiked) had lower *mcr*A copies, and was dominated by Methanobacteriaceae, typically restricted to CO_2_ reduction methanogenic pathways (Liu and Whitman, 2008; De Vrieze et al., 2012). This pattern of lower methanogen activity at higher TYP concentrations could have been due to a lag in available metabolites for methanogens, a pattern supported by the temporal lag seen in CH_4_ production with increasing TYP treatment concentrations over the 150-day incubation (Supplementary Figure S3). The delay in the onset of methanogenesis could have been be due to competition by other microbial guilds (e.g., sulfate reducers also capable of H^+^ or acetate utilization) dominating when larger amounts of fresh, less decomposed, litter were present. A similar temporal pattern was previously reported in an experiment examining the degradation of switchgrass in a rumen environment, where 13% of the biomass was degraded in the first 30 min, followed by 3.5 h period of reduced biomass degradation where methanogens increased in cell number threefold (Piao et al., 2014). Therefore, our data demonstrate the dependency of methanogens on syntrophic bacterial decomposers for metabolites for methanogenesis. Further, here we show how certain plant litters (DEC and CON) can inhibit decomposition of plant matter by syntrophs and subsequently methanogens, resulting in overall lower metabolic rates relative to more labile plant litters (TYP; Figure 1).

## Conclusion

Our study demonstrates how lake sediment microbial communities are intertwined in the processing and fate of terrestrial organic inputs. We observed changes in the composition, richness and abundance of bacteria, fungi, and methanogens in our sediments amended with different plant litters. The bacterial orders that dominated the different litters correlated with the polyphenol content, showing a specialization of the community to specific litter types. In a broader ecological context, our study provides evidence for the mechanism by which sediment CH_4_ production is modulated by plant litter compositions through changing microbial community compositions. We also showed that fungi can be important decomposers in anaerobic lake sediments with high polyphenolic content plant litter, with corresponding dominance of a specialized polyphenol decomposer, *Phlebia* spp. Our work indicates that the polyphenol biochemistry during decomposition dictates the microbial communities responsible for greenhouse gas production, directing the fate of carbon in lakes.

## Author Contributions

KY, EE, MC, AT, NB, and NM conceived the study. KY, EE, and MC collected the data. KY analyzed the data and wrote the manuscript with input from all authors.

## Conflict of Interest Statement

The authors declare that the research was conducted in the absence of any commercial or financial relationships that could be construed as a potential conflict of interest.
