# Supplementary material for: Plant Litter Type Dictates Microbial Communities Responsible for Greenhouse Gas Production in Amended Lake Sediments
Source: Front Microbiol. 2018 Nov 6;9:2662. doi: 10.3389/fmicb.2018.02662 (PMC6232422; doi:10.3389/fmicb.2018.02662)
Supplement: Supplementary file 1 [file Data_Sheet_1.PDF]

Supplementary Figures:

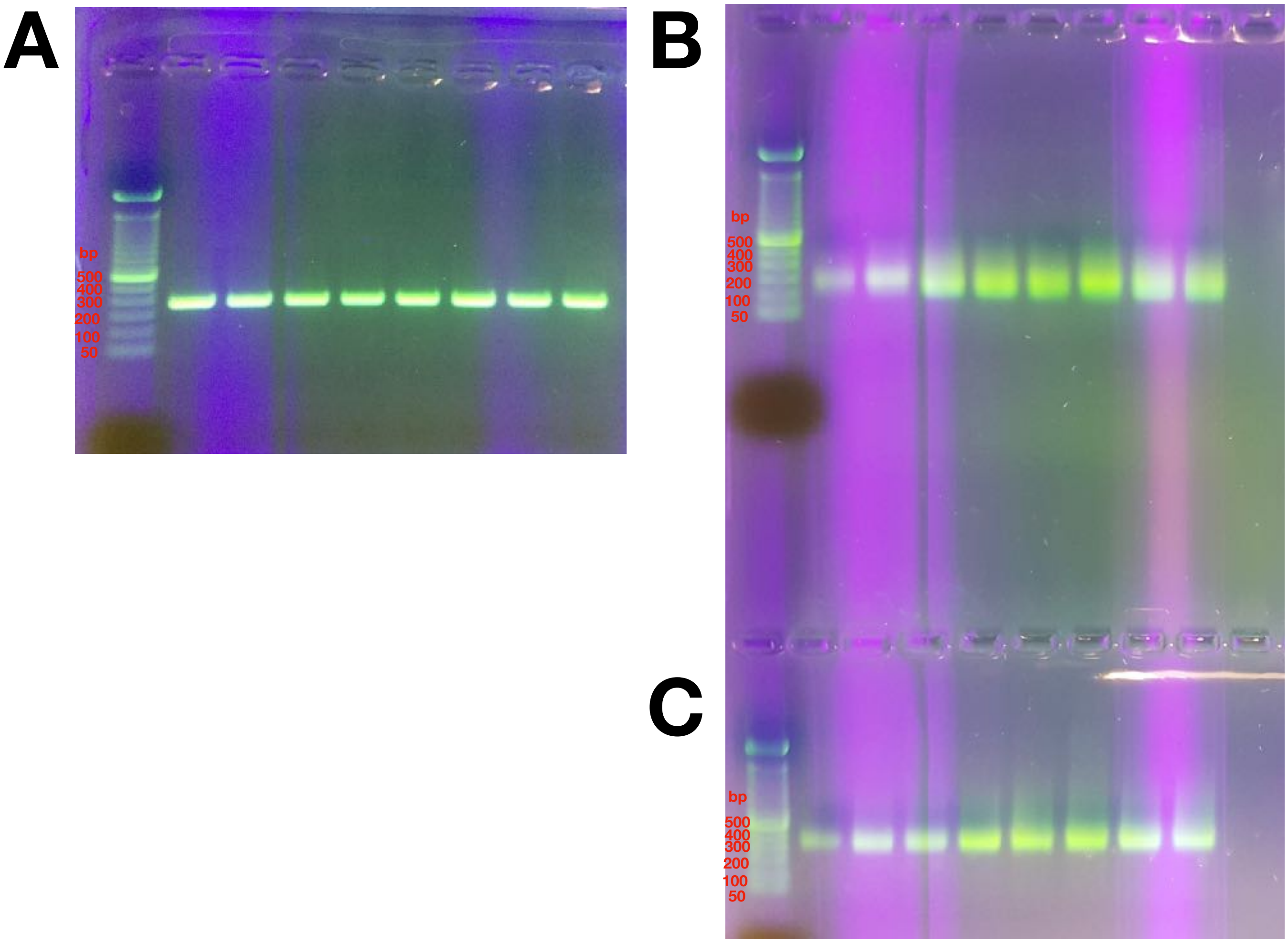


Supplementary Figure 1: A select few samples were chosen to be run an a gel after qPCR to ensure proper amplification was achieved. Above are the 1.5% agarose gel images of the qPCR products from two each of the 10% samples for each plant litter type, in order left to right CON, DEC and TYP. The last two bands are from the coniferous the and deciduous litter controls respectively. Gel A is of the *mcr*A, gel B is 16s rDNA and gel C is of the 18s rDNA data.As there were no anomalies in the qPCR results, dissociation curve, or gel results, no further samples were run on an agaraose gel.

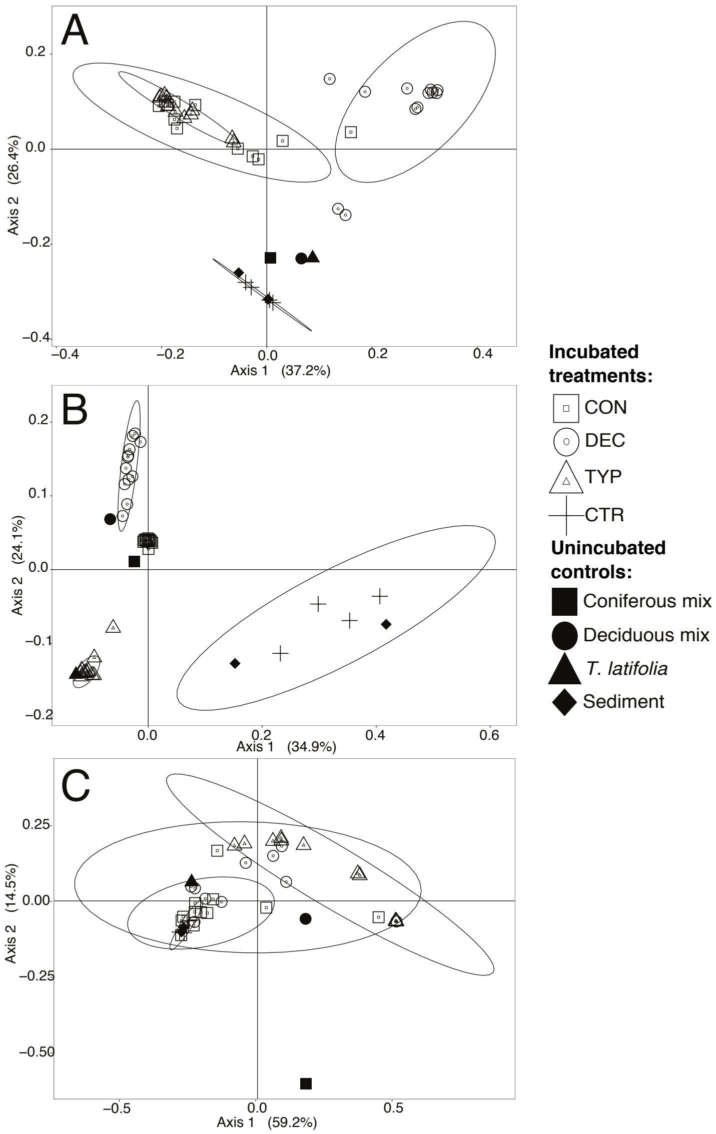


Supplementary Figure 2: PCoAs of weighted UniFrac distances using relative abundances for the respective microbial communities; bacteria (A; 5565 OTUs), fungi (B; subset of 128 OTUs), and methanogens (C; 43 OTUs), across all treatment concentration. Ellipses represent 95% confidence intervals which were calculated for each incubated treatment. The proportion of variation explained by each axis is given in parentheses.

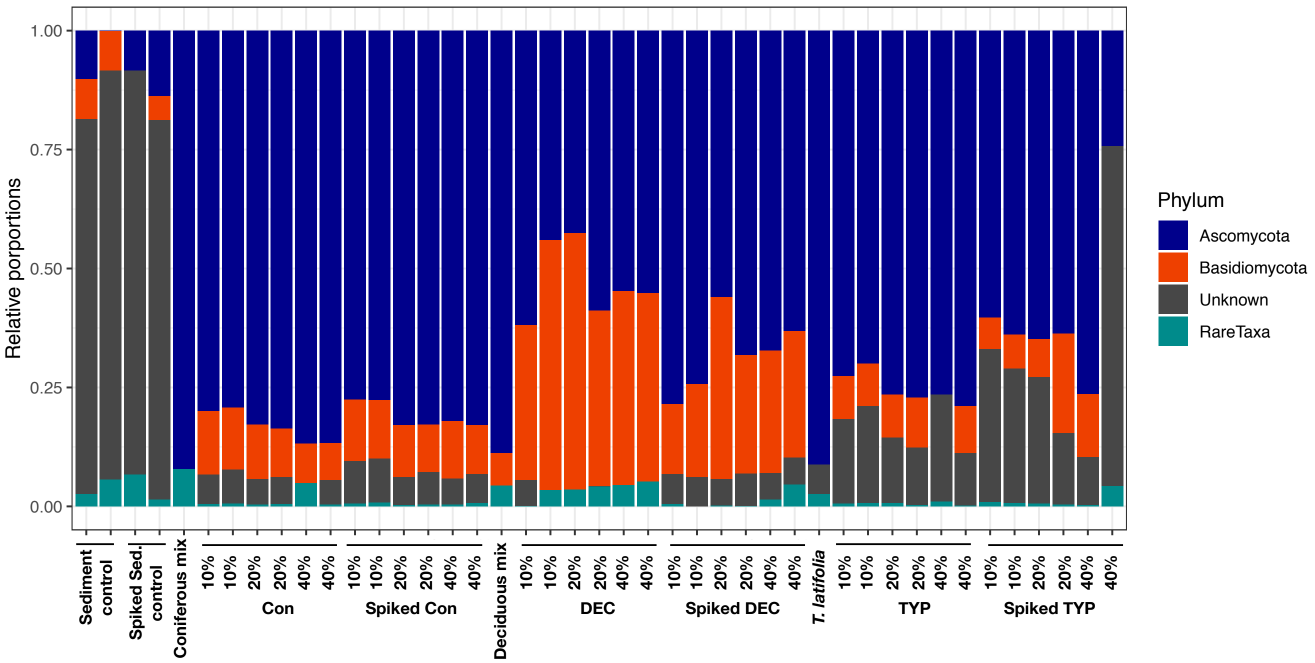


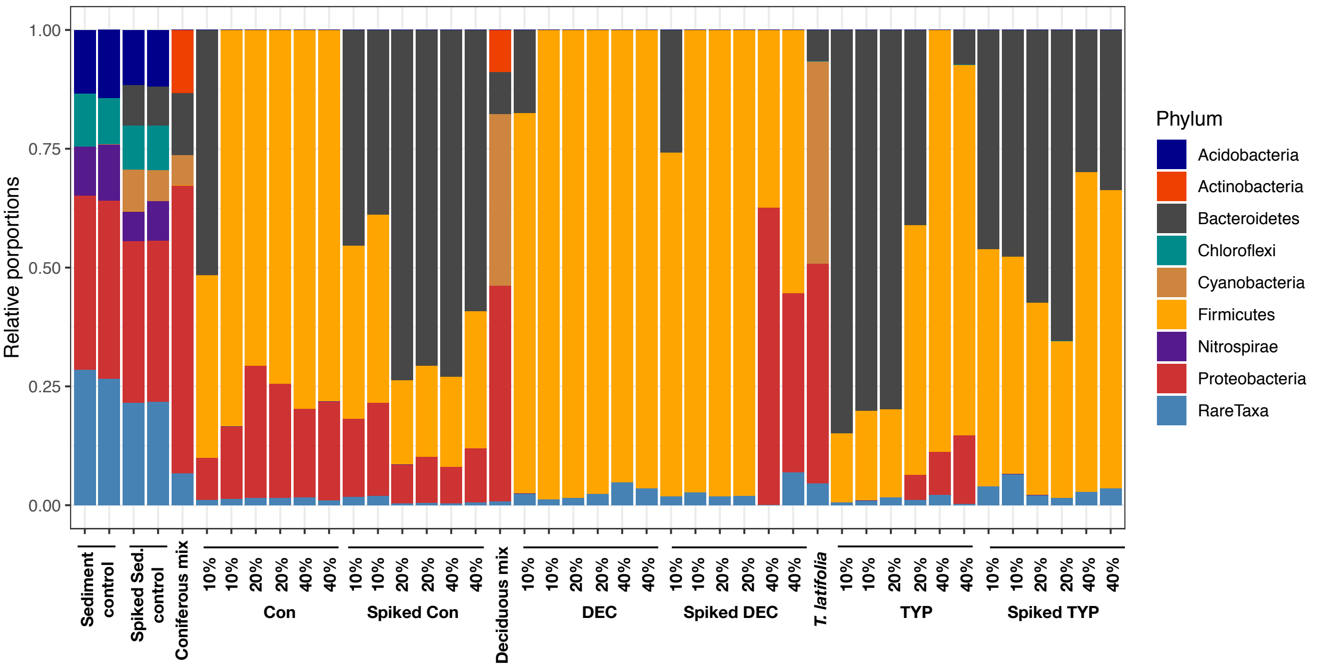


Supplemental Figure 3: Relative abundance of each of the sediment controls, litter controls and their respective litter treatments. The top panel is the bacterial phyla from the 16s rDNA data, and the bottom panel is the fungal phyla from the ITS sequencing data. Values are the average of replicate sequencing runs. All phyla that were present as <5% relative abundance was grouped as Rare Taxa

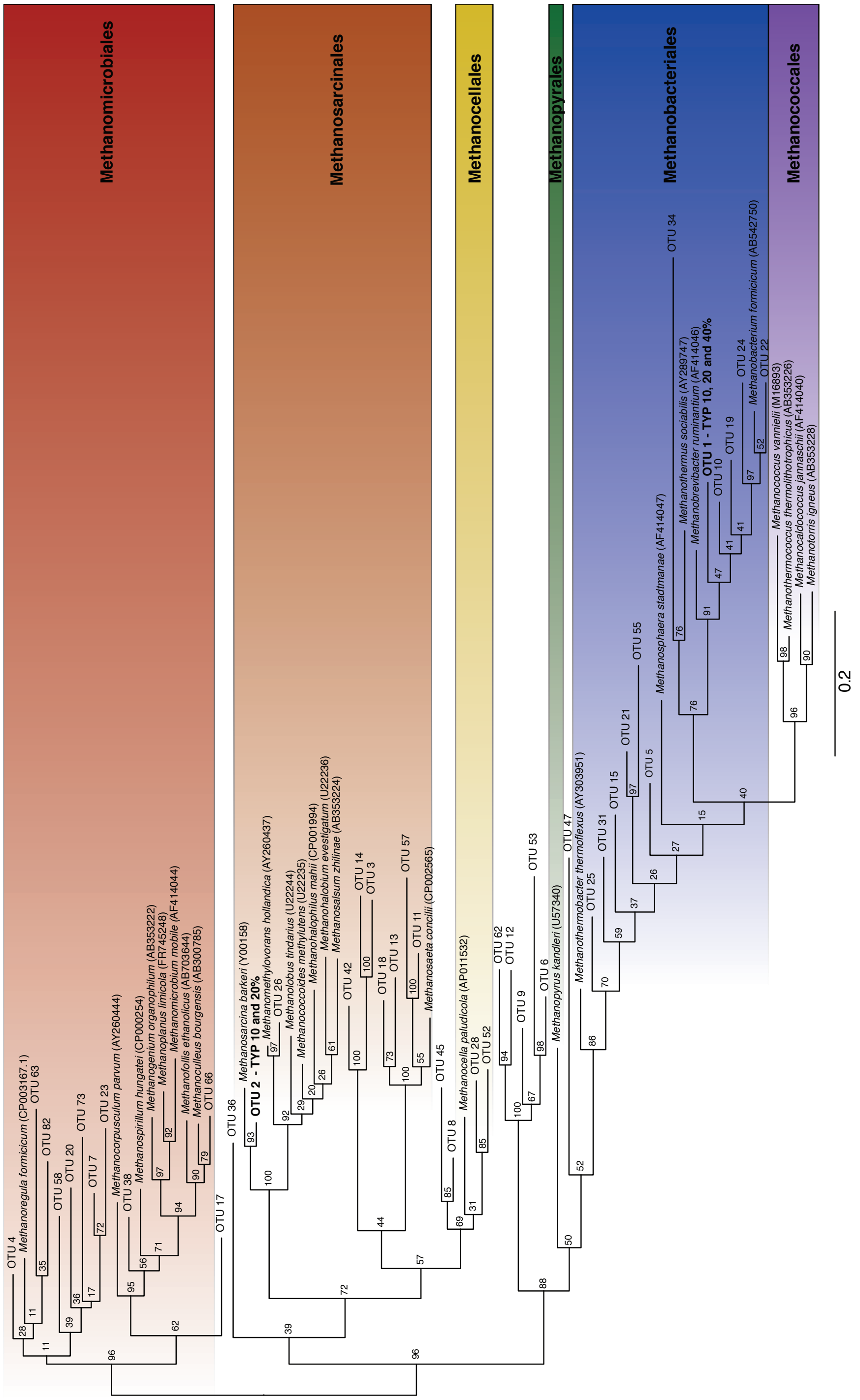


Supplementary Figure 4: Maximum-likelihood phylogenetic tree of the mcrA gene containing the 43 OTUs found in all treatments. Node values are from 1000 bootstrap replicates and branches were arranged and highlighted by methanogen orders. Reference sequences from described species were selected to represent the orders of methanogens and were obtained from GenBank and their tip labels contain their accession number.

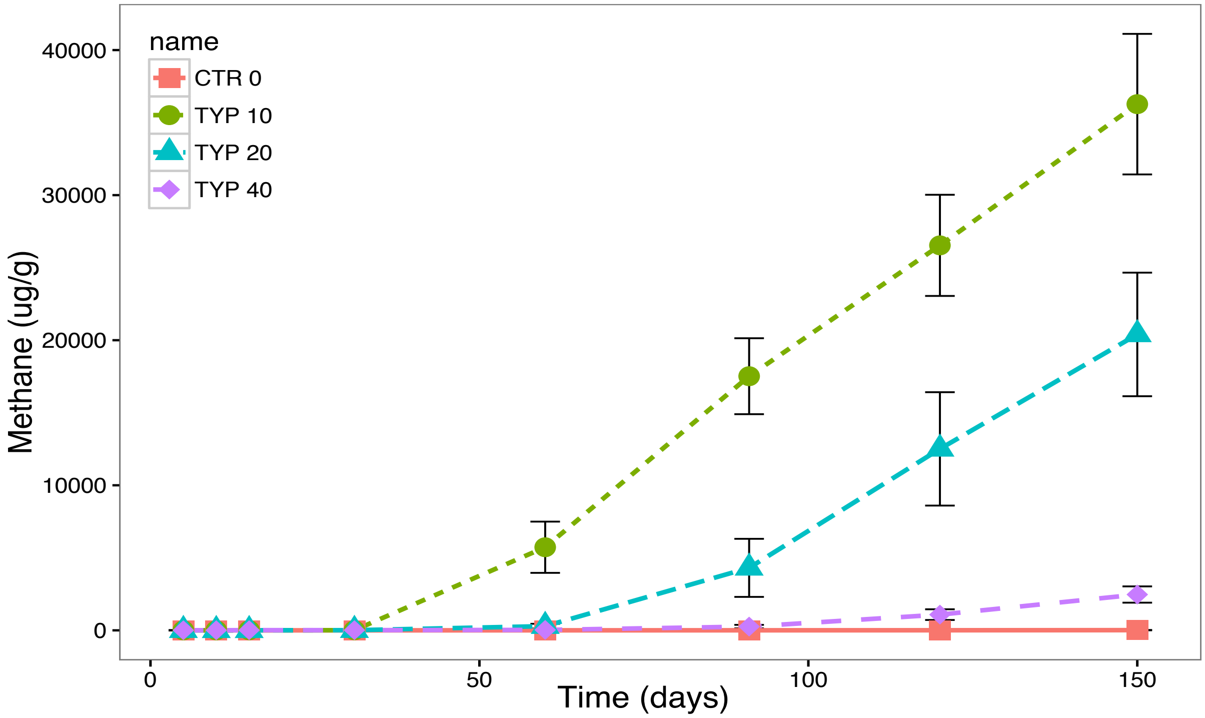


Supplementary Figure 5: Cumulative production of $CH_4$ over time for the un-spiked TYP treatment, displayed by percent organic matter. Points are means with ±SE for µg methane in g per dry weight of sediment. CTR represents the un-spiked sediment control.

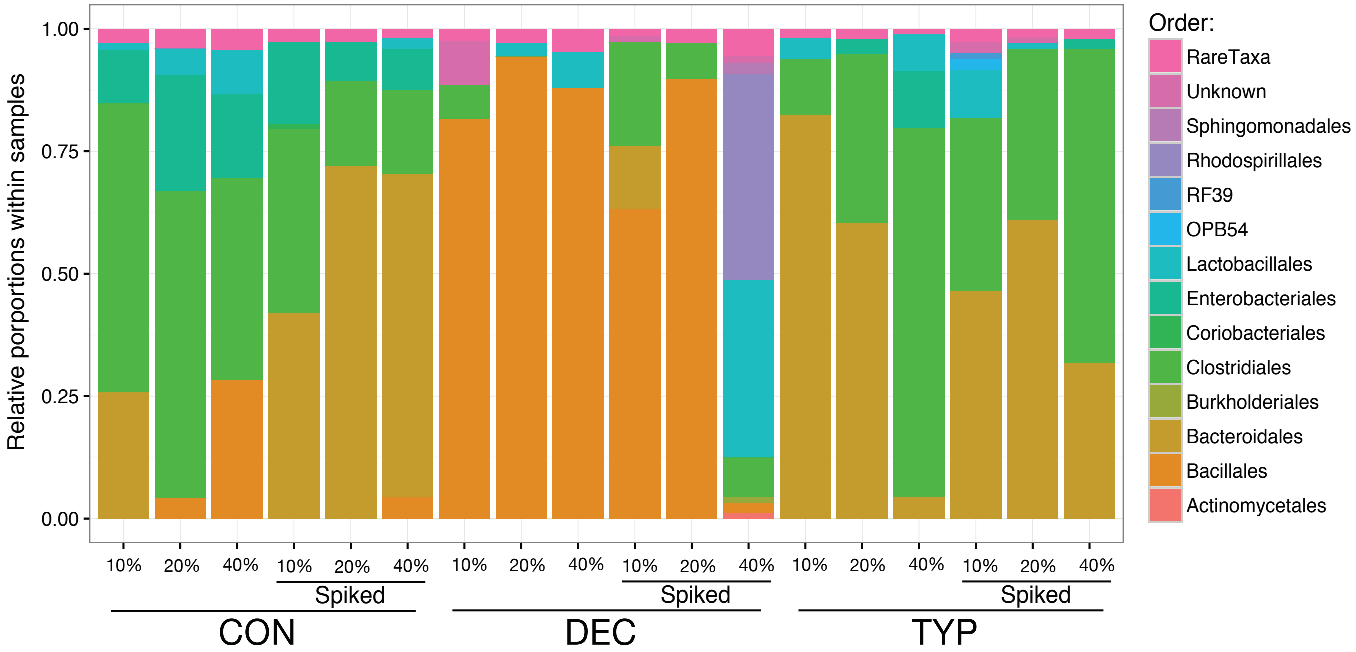


Supplementary Figure 6: Relative abundance of each litter sample and concentration for bacterial orders. Values are the average of replicate sequencing runs. All Orders that were present as <1% relative abundance were grouped as Rare Taxa.

Supplemental Tables

Supplementary Table 1: Mean pH values from incubated treatments. Values in brackets below averages are standard error values.

| Treatment | Unspiked | Spiked |
|---|---|---|
| CON | 4.75<br>(0.12) | 4.38<br>(0.27) |
| DEC | 4.20<br>(0.10) | 3.63<br>(0.25) |
| TYP | 5.37<br>(0.23) | 5.20<br>(0.32) |
| CTR | 6.94<br>(0.24) | 4.93<br>(0.24) |
